# Human Papillomavirus Vaccination in South Africa: Programmatic Challenges and Opportunities for Integration With Other Adolescent Health Services?

**DOI:** 10.3389/fpubh.2022.799984

**Published:** 2022-01-31

**Authors:** Edina Amponsah-Dacosta, Ntombifuthi Blose, Varsetile Varster Nkwinika, Viola Chepkurui

**Affiliations:** ^1^Vaccines for Africa Initiative, Faculty of Health Sciences, School of Public Health and Family Medicine, University of Cape Town, Cape Town, South Africa; ^2^Division of Epidemiology and Biostatistics, Faculty of Health Sciences, School of Public Health and Family Medicine, University of Cape Town, Cape Town, South Africa; ^3^Department of Virological Pathology, Sefako Makgatho Health Sciences University, Pretoria, South Africa; ^4^Department of Virological Pathology, South African Vaccination and Immunisation Centre (SAVIC), Sefako Makgatho Health Sciences University, Pretoria, South Africa

**Keywords:** adolescent, cervical cancer, HIV, human papillomavirus, South Africa, sub-Saharan Africa, vaccine

## Abstract

Compared to other regions of the world, sub-Saharan Africa has made limited progress in the implementation and performance of nationwide human papillomavirus (HPV) vaccination programmes. Without urgent intervention, this will serve to undermine cervical cancer elimination efforts in this region. The primary intent of this narrative review is to highlight the programmatic successes and challenges of the school-based HPV vaccination programme in South Africa since its inception in 2014, with the aim of contributing to the evidence base needed to accelerate implementation and improve programme performance in other sub-Saharan African countries. As of 2020, the proportion of adolescent girls aged 15 years who had received at least one dose of the HPV vaccine at any time between ages 9–14 years was 75%, while 61% had completed the full recommended two-dose schedule. This gives some indication of the reach of the South African HPV vaccination programme over the past 6 years. Despite this, vaccine coverage and dose completion rates have persistently followed a downward trend, slowing progress toward attaining global elimination targets. There is evidence suggesting that declining public demand for the HPV vaccine may be a result of weakening social mobilization over time, inadequate reminder and tracking systems, and vaccine hesitancy. Another concern is the disproportionate burden of HPV and HIV co-infections among adolescent girls and young women in South Africa, which predisposes them to early development of invasive cervical cancer. Moving forward, national policy makers and implementers will have to explore reforms to current age eligibility criteria and vaccine dose schedules, as well as implement strategies to support vaccine uptake among populations like out-of-school girls, girls attending private schools, and HIV positive young women. Additional opportunities to strengthen the South African HPV vaccination programme can be achieved by scaling up the co-delivery of other adolescent health services such as comprehensive sexual and reproductive health and rights education, deworming, and health screening. This calls for reinforcing implementation of the integrated school health policy and leveraging existing adolescent health programmes and initiatives in South Africa. Ultimately, establishing tailored, adolescent-centered, integrated health programmes will require guidance from further operational research.

## Introduction

Cervical cancer is the fourth most frequently diagnosed cancer and the fourth leading cause of cancer-related death among women worldwide (1). The brunt of the global disease burden is borne by countries in Asia, and in sub-Saharan Africa where concerning trends in increasing cervical cancer incidence rates have been reported (2). In South Africa, cervical cancer incidence and mortality rates are among the highest in sub-Saharan Africa. Of the 117,316 new cervical cancer cases and 76,745 deaths recorded within the African region in 2020, 10,702 cases and 5,870 associated deaths were reported from South Africa alone (3). By contrast, a total of 4,664 deaths due to female breast cancer were recorded in that same year, making cervical cancer the leading cause of cancer-related death among women in South Africa (3). Persistent infection with oncogenic types of the human papillomavirus (HPV) acquired through sexual contact is the primary risk factor for about 90% of cervical cancer cases (4). Another important factor is co-infection with the human immunodeficiency virus (HIV), which has been associated with an increased risk of persistent HPV infection and rapid progression to invasive cervical cancer. In fact, reports from sub-Saharan Africa suggest that women living with HIV are more likely to develop cervical cancer with poorer outcomes compared to their HIV uninfected counterparts (5–7). Aside from having the highest number of people (7.8 million) living with HIV in the world, of which 62% are adolescent girls and young women (AGYW) >15 years of age, estimates from South Africa also show a significantly higher prevalence of infection with high-risk HPV types among AGYW (18–25 years) who are HIV infected (72.7%) compared to those who are HIV uninfected (34.1%) (8, 9).

Recognizing the devastating impact of cervical cancer on public health, the World Health Assembly passed a resolution in August 2020 to accelerate the elimination of cervical cancer, calling for all member states to adopt multi-sectoral and integrated healthcare approaches aimed at achieving the following triple-intervention coverage targets by 2030; (i) vaccination: 90% of adolescent girls fully vaccinated with the HPV vaccine by the age of 15 years, (ii) screening: 70% of women screened using a high-performance test by the age of 35, and again by the age of 45 years, and (iii) treatment: 90% of women with cervical pre-cancer treated and 90% of women with invasive cervical cancer managed (10). Currently, the pooled uptake of cervical cancer screening services in sub-Saharan Africa is estimated at 12.87% (95% CI: 10.20–15.54) and requires drastic up-scaling if global elimination targets are to be attained (11). In South Africa, the national cervical cancer prevention and management programme is guided by the 2017 Cervical Cancer Prevention and Control Policy (12) which aligns with the current global framework for elimination of cervical cancer and makes careful consideration for the local context including the co-burden of HIV infection. Coverage of cervical cancer screening services in South Africa is more encouraging than regional estimates, with the prevalence of Papanicolaou smear test among women aged >30 years reaching 52%. Despite this, significant socio-economic disparities exist in accessing these screening services (13). Consequently, a substantial proportion of cervical cancer cases are diagnosed when the disease is at an advanced stage, at which point treatment options are limited and treatment outcomes are bleak. Fortunately, most cervical cancer cases are entirely preventable by vaccinating adolescent girls before sexual debut and first exposure to HPV infection. The World Health Organization (WHO) recommends a two-dose schedule of the HPV vaccine administered 6–12 months apart to girls aged 9–14 years as the primary target for prevention of cervical cancer (14). For girls >14 years of age, the WHO recommends a three-dose schedule of the vaccine. Vaccination against HPV infection is one of the most efficacious and cost-effective interventions available, and when effectively incorporated alongside screening programmes, provides equitable solutions for comprehensive cervical cancer prevention and control.

**Figure 1:**
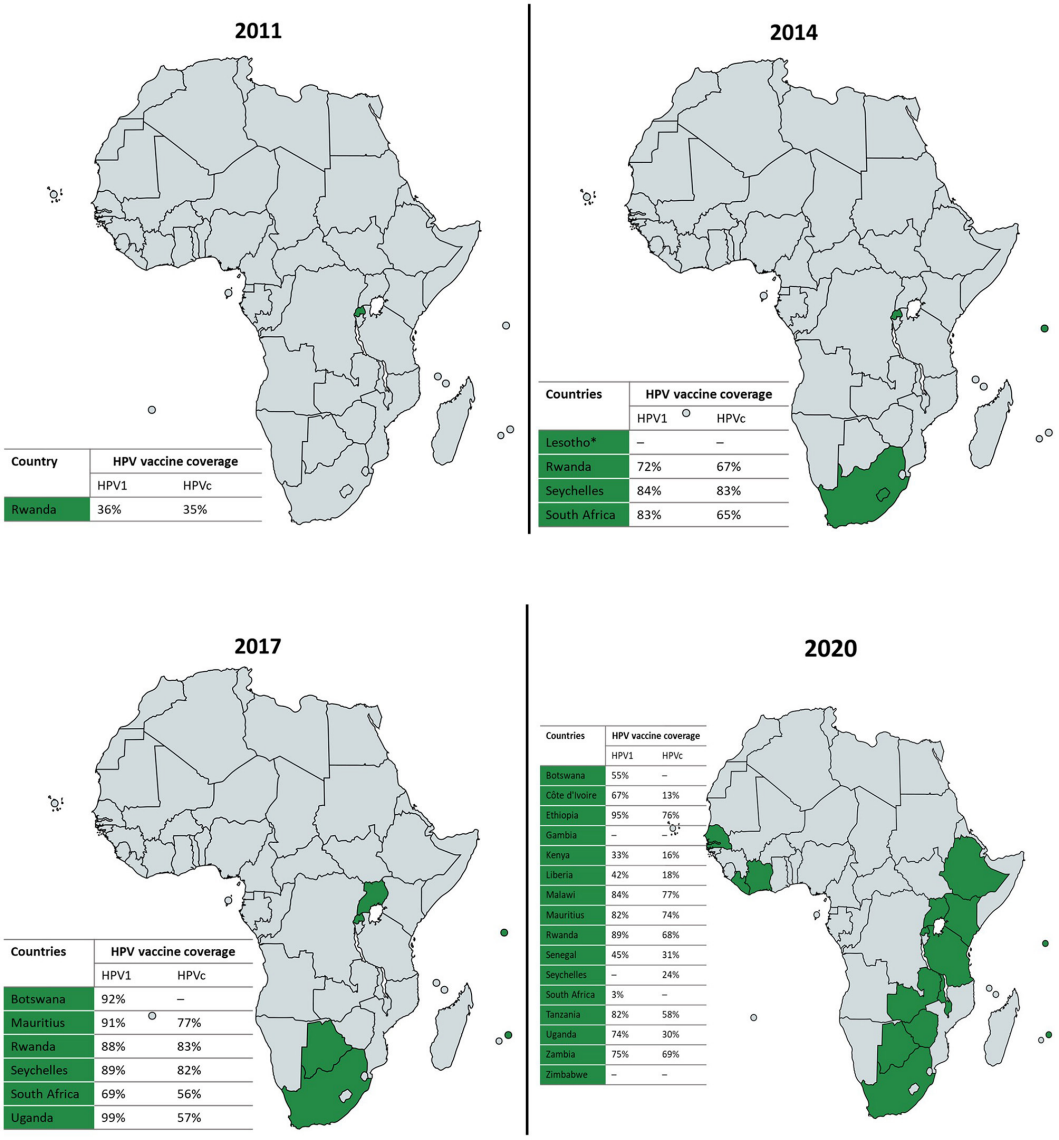
Progress of nationwide HPV vaccine introduction and coverage in sub-Saharan Africa, 2011–2020 (16, 17). Where available, national HPV vaccine coverage data for the first (HPV1) and last (HPVc) doses per each reporting year is presented in the accompanying tables. *Lesotho implemented nationwide HPV vaccination in 2012 but stopped in 2015; and is projected to have the HPV vaccine back on the national routine immunization schedule by 2022. Maps created in https://mapchart.net.

Three licensed prophylactic HPV vaccines – the bivalent, quadrivalent and nonavalent HPV vaccines – with excellent safety profiles and relatively similar effectiveness in preventing cervical cancer are currently available for use. As of June 2020, the proportion of countries that had introduced the HPV vaccine partially or nationwide as part of their national immunization programme per region was as follows: 85% in the Americas, 77% in Europe, 56% in Oceania, 40% in Asia, and finally 31% in Africa (15). Where the vaccine has been introduced, coverage of the last dose ranges from 20% (95% CI: 5–39%) in sub-Saharan Africa to 77% (95% CI: 67–79%) in Australia and New Zealand (15). These coverage estimates reflect significant regional and income level disparities in the introduction and performance of HPV vaccination programmes, undermining elimination efforts in the most affected parts of the world. While HPV vaccines have been widely available in high resource regions since 2006, the first nationwide roll-out of HPV vaccination in sub-Saharan Africa was in 2011 (16). The progress of nationwide HPV vaccine introduction and coverage across sub-Saharan Africa between 2011 and 2020 is presented in Figure 1. Sub-Saharan African countries have made limited progress in adopting the HPV vaccine into national immunization programmes and have generally achieved suboptimal vaccine coverage, although significant inter-country variations exist. The delay in widely adopting and sustaining nationwide HPV vaccination programmes has been attributed to the substantial financial and technical resource requirements linked with introducing the vaccine, while programmatic barriers and broader health systems constraints have been cited as determinants of suboptimal vaccine coverage in sub-Saharan Africa (18). Unfortunately, recent global HPV vaccine supply shortages and the ongoing coronavirus disease (COVID-19) pandemic caused by the novel severe acute respiratory syndrome coronavirus 2 have further hampered progress in this region (19, 20).

In South Africa, the bivalent and quadrivalent HPV vaccines were first approved for use in 2008, although only available at a cost through the private health sector at the time. In April 2014, South Africa joined countries like Rwanda and Seychelles as one of the first few sub-Saharan African countries to roll-out nationwide HPV vaccination, offered free of charge to grade 4 adolescent girls ≥9 years of age, attending public schools across the country (21). The school-based vaccination programme was determined to be the most cost-effective and successful model for delivering the HPV vaccine to eligible adolescent girls in the South African context (22, 23).

It is also worth noting that adolescents in South Africa constitute about 19% of the overall population (24). The burden of disease among this adolescent population has been recognized as a major cause for concern, with poor sexual and reproductive health outcomes including unintended and unsupported teenage pregnancies, as well as HIV and AIDS, and non-communicable diseases like cancer, diabetes, and hypertension, being identified as key threats to their wellbeing (24). Supporting their wellbeing and transition into adulthood will require a comprehensive and tailored package of care. Typically, adolescents have limited contact with the health system despite having significant healthcare needs. In addition, adolescent and school-based health services may not always be responsive to the expressed healthcare needs of this population (25, 26). Given its longstanding nationwide HPV vaccination programme, South Africa presents a unique case for critically assessing programmatic successes and challenges, which could inform recommendations for future and existing HPV vaccination programmes within sub-Saharan Africa. Thus, to contribute to the evidence base on prioritizing adolescent health, we review the progress of the HPV vaccination programme to date and the scope of adolescent and school-based health services in South Africa. In addition, we explore opportunities for effectively delivering adolescent health services alongside the HPV vaccination programme in South Africa.

## Trends in HPV Vaccine Coverage Among Adolescent Girls in South Africa, 2014–2020

**Figure 2:**
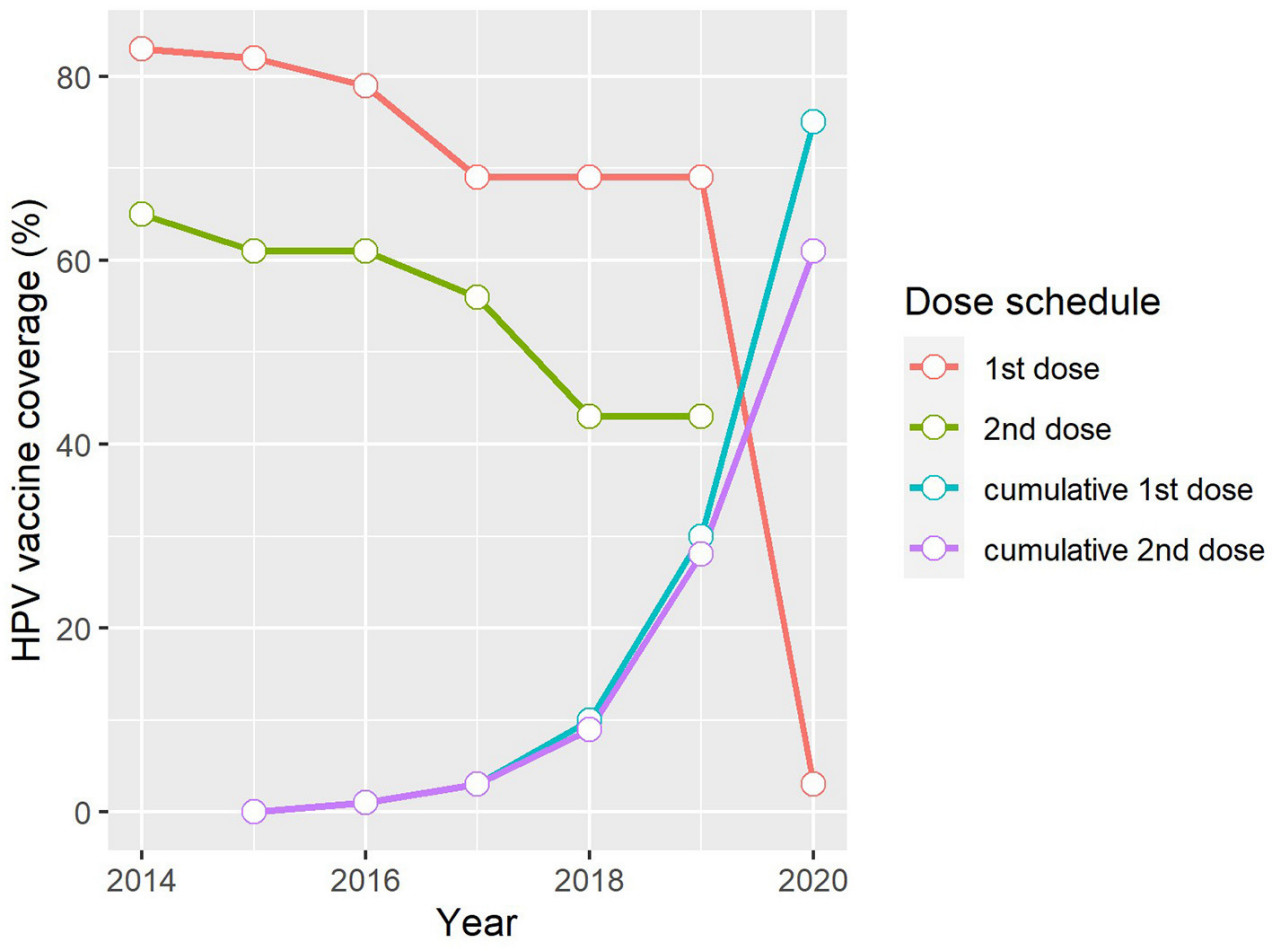
Trends in HPV vaccine coverage in South Africa, 2014–2020 (17). 1st dose, Proportion of the target population who received the first dose of the HPV vaccine in the reporting year. 2nd dose, Proportion of the target population who received the last dose of the HPV vaccine in the reporting year. Cumulative 1st dose, Proportion of the population turning 15 years in the reporting year who received at least one dose of the HPV vaccine any time between ages 9–14 years. Cumulative 2nd dose, Proportion of the population turning 15 years in the reporting year who received the full recommended schedule of the HPV vaccine any time between ages 9–14 years.

Of the 503,507 eligible grade 4 girls aged ≥9 years attending South African public schools in April 2014, 419,520 were vaccinated with the first dose of the bivalent HPV vaccine during the first round of the nationwide HPV vaccination programme, achieving a high coverage rate of 83% (17). The second round of vaccinations administered 6 months later reached 65% of the overall proportion of eligible girls. Since then, national HPV vaccine coverage and dose completion rates have followed a downward trend (Figure 2). This contrasts with the upward trend in vaccine coverage observed in Rwanda where a nationwide HPV vaccination programme has been in existence for close to a decade (27), as well as in Seychelles, where a school-based programme was also first introduced in 2014 (Figure 1) (16, 17). Notably, variations exist in HPV vaccine coverage rates across provinces and within districts in South Africa. In an assessment of the first round of the vaccination programme, Delany-Moretlwe et al. (21) reported isolated pockets of low vaccine coverage in the country. Two sub-districts in the KwaZulu Natal and Mpumalanga provinces were reported to have achieved coverage rates as low as 40 and 43%, respectively.

The disparity in coverage rates between the first and last vaccine doses is also worth noting and suggests an increasing dropout rate over time, ranging from 18% in 2014 to as high as 26% in both 2018 and 2019 (Figure 2). The challenges experienced in maintaining high HPV vaccine coverage and low dropout rates is not unique to the South African context but is consistent with reports from other low- and middle- income countries (LMICs) where mean dropout rates have been estimated at 18%, which is relatively high when compared to the 11% estimated for high-income countries (15). Factors suggested to influence completion of the HPV vaccine regimen among adolescent girls in LMICs include inadequate reminder strategies for the last dose, poor tracking systems required to identify those who miss their doses, weakening social mobilization efforts over time, and inadequate refresher training for vaccinators prior to rolling out the second round of vaccinations (28).

Where South Africa's progress toward achieving the 2030 vaccine coverage target is concerned, the WHO-UNICEF cumulative data collected by 2020 show that the proportion of adolescent girls aged 15 years who had received at least one dose of the HPV vaccine at any time between ages 9–14 years was 75% while 61% had received the full recommended schedule (Figure 2). It is important to note that national coverage data do not include those from the private sector as adolescent girls attending private schools do not receive the vaccine as part of the school-based programme. Instead, this population group is encouraged to access the vaccine through their health service providers. The direct cost to the national government for a single dose of the bivalent HPV vaccine is R341.76 ZAR ($23 USD) per learner in public schools (average exchange rate in 2021: R14.59 ZAR). In the private health sector where both the bivalent and quadrivalent vaccines are available, the current price of either vaccine type averages at just over R900 ZAR ($62 USD) per dose (excluding the service cost for administering the vaccine) and may be partially or fully covered by private medical insurance. In the absence of nationwide estimates of HPV vaccine coverage rates in the private school sector, a cross-sectional Facebook survey conducted by Milondzo et al. (29) which investigated HPV vaccination practices of caregivers of grade 4–7 girls aged ≥9 years attending private schools in South Africa, found that only 19.4% (80/413) of these girls had received at least one dose of the HPV vaccine.

Overall, coverage rates over the past 6 years of the nationwide HPV vaccination programme in South Africa are lower than anticipated, given the high uptake rates (>90%) reported during early demonstration projects and feasibility studies (22, 30). This may suggest the emergence of critical gaps – including logistical and operational challenges – as the vaccination programme matures. Undoubtedly, the COVID-19 pandemic has exacerbated these challenges. While coverage data for the last dose of the HPV vaccine in 2020 is currently not available via the WHO-UNICEF reporting system, that of the first dose, which is estimated at a dismal 3% (Figure 2) (17), gives some indication of the extent of the disruption caused by the COVID-19 pandemic, largely owing to lockdowns and school closures instituted to mitigate the spread of the disease. An improved understanding of the programmatic successes and challenges over the past 6 years will help inform tailored interventions to scale up HPV vaccine uptake and completion rates in South Africa.

## Factors Influencing the Performance of the South African HPV Vaccination Programme

### Supply-Side Determinants of Programme Performance

The successful implementation of the nationwide HPV vaccination programme in South Africa has been largely attributed to the strong political commitment, intersectoral collaboration among the National Departments of Health, Basic Education and Social Development, careful planning and coordination at provincial and district levels, and strong leadership from the National Department of Health (21). This strong governance and high-level commitment have also been major drivers of sustainability of the vaccination programme over time. What is emerging as a critical challenge to the programme as it matures is the downward trend in vaccine uptake and dose completion rates among eligible adolescent girls. The evidence base on factors influencing HPV vaccine uptake in South Africa is heavily focused on demand-side determinants such as end-user knowledge, attitudes, and practices, which are discussed further on. In addition, much of the available evidence on the determinants of vaccine uptake was either generated through early feasibility studies, or immediately after the nationwide roll-out of the vaccination programme. While the HPV vaccination programme has only been in existence for 6 years, the field's rapid evolution means that determinants of vaccine uptake and dose completion are bound to change over time and should be comprehensively researched and monitored.

On the supply side, factors such as appropriate vaccine dose schedules, eligibility, cost, vaccine delivery strategies, vaccine availability and accessibility, partnerships with key stakeholders, quality of health infrastructure, and capacity of vaccinators including staffing, capability, and level of knowledge, can all influence HPV vaccine uptake and completion rates. Although the bivalent and quadrivalent HPV vaccines were initially licensed as three-dose vaccines, a two-dose schedule was widely adopted following demonstration of non-inferior immunogenicity between both schedules (14, 31). The two-dose schedule was also demonstrated to achieve higher vaccine coverage with lower dropout rates. A review (31) of preliminary evidence now suggests that a single dose of the HPV vaccine may provide protection against persistent HPV infection and thereby reduce the incidence of HPV-related sequelae. Furthermore, vaccination with a single dose of the HPV vaccine has the potential to achieve higher coverage and completion rates at a relatively lower cost compared to the current two-dose schedule (31). In South Africa, modeling analysis has been used to demonstrate the effectiveness of single dose nonavalent HPV vaccination in achieving a considerable reduction in the incidence of HPV infection and cervical cancer, as well as in the associated mortality rates in a setting where both HPV and HIV infections are endemic (32). Moreover, the findings of this modeling study appear to be generalizable to the bivalent HPV vaccine which is currently in use as part of the nationwide HPV vaccination programme. As the evidence base on the immunogenicity of a single dose HPV vaccine regimen develops, policymakers may be able to adopt an alternative vaccination strategy that offers optimal vaccine coverage and completion rates without compromising on vaccine effectiveness.

Policy decisions on the eligibility for the HPV vaccine is another critical supply-side factor as limited and missed opportunities for vaccination could contribute to suboptimal vaccine uptake and compromise elimination efforts. There have been calls to expand the reach of the South African HPV vaccination programme given the unique epidemiology of HPV infection and co-burden of other sexually transmitted infections (including chlamydia, genital herpes, gonorrhea, mycoplasma genitalium, and trichomoniasis) among AGYW >15 years of age (33). Several studies (34–37) conducted among AGYW >15 years in South African universities either prior to, or following the nationwide roll-out of the HPV vaccination programme have generally reported suboptimal levels of knowledge about HPV, cervical cancer, and the HPV vaccine within this population, which contributes to low-risk perceptions, and risky sexual behaviors and practices. Despite this, female university students consistently demonstrate high levels of HPV vaccine acceptance and willingness to vaccinate if the HPV vaccine were made available to them (34–37). The increased risk of HPV infection makes AGYW >15 years good candidates for targeted interventions aimed at accelerating the prevention of new cervical cancer cases while the observed positive attitude to the HPV vaccine could suggest optimal coverage rates should the vaccination programme be extended to include this population. Unfortunately, expanding the target population to include AGYW >15 years of age will incur substantial costs, which may not be ideal considering that the South African government fully finances its own vaccination programme without donor support. Policy reforms aimed at providing AGYW >15 years of age with low-cost HPV vaccination through the public health sector may be an alternative option worth exploring in the context of optimal vaccine supply. For LMICs, it has been proposed that expanding the reach of lifesaving HPV vaccines to all AGYW can be achieved through production of low-cost generic HPV vaccines, adopting single-dose HPV vaccine schedules, and leveraging the HPV vaccine delivery platform to deliver other adolescent health services, including other adolescent vaccines (38). On 14 October 2021, a new bivalent HPV vaccine manufactured by Xiamen Innovax Biotech Co. Ltd. received prequalification from the WHO (39, 40). As the fourth HPV vaccine to receive WHO prequalification, this could be a major step toward easing HPV vaccine supply and affordability challenges faced by most LMICs. While the possibility of additional low-cost generic HPV vaccines and single-dose vaccine schedules are being explored, stopgap solutions should be implemented to address the declining HPV vaccine uptake and dose completion rates among eligible girls in South Africa. This may include intensifying reminder strategies for the last dose, improving tracking systems to identify girls who miss their last dose, including those who may have transitioned between the public and private school sectors, and strengthening catch-up vaccination programmes to reach those who may have missed their scheduled doses due to absenteeism, as well as out-of-school girls.

In terms of HPV vaccine availability and accessibility in South Africa, providing free HPV vaccination to eligible girls continues to be a major driver of vaccine uptake. According to the 2019 School Realities Report by the Department of Basic Education (41), of the 548,112 adolescent girls in grade 4, 520,184 (94.9%) attended public schools, while 27,928 (5.1%) were in the private school sector. This gives some indication of the potential reach of the public school-based vaccination programme. On the other hand, the health facility-based vaccination strategy which is targeted at adolescent girls in private schools has been identified as a barrier to vaccine uptake (29). For those who can access the vaccine through the private health sector, medical insurance providers may not always be prepared to cover the full cost of vaccination. Furthermore, waiting for adolescent girls and their parents or caregivers to make the decision to access the HPV vaccine or return to health facilities for their last dose may lead to lower uptake and completion rates compared to the school-based strategy. Adolescents and their parents or caregivers are reliant on the information provided by their healthcare providers in making decisions about their health. This makes the level of knowledge and attitude among healthcare providers about vaccine messaging, as well as their recommendations on reliable sources of information, and who they target for communicating about the HPV vaccine, critical to effective vaccine demand generation. An early feasibility study found that healthcare providers were generally less well informed about HPV infection, its link with cervical cancer, and the risks and benefits of the HPV vaccine, which tended to fuel their reluctance to recommend or administer the vaccine to their clients (42). With the nationwide roll-out of the HPV vaccination programme over the past 6 years and the availability of training for vaccinators and healthcare providers in general, improvements in vaccine recommendations and practices can be anticipated. Ultimately, further engagements with stakeholders from the private school and health sectors to co-develop interventions, including educational campaigns and reminders aimed at improving HPV vaccine uptake are needed, although these will have to be informed by further research on the barriers and facilitators of HPV vaccine acceptance and uptake among girls in private schools.

Given the current context, it is worth highlighting how South Africa has approached the school-based HPV vaccination programme during the COVID-19 pandemic. Intermittent school closures in response to multiple waves of the COVID-19 pandemic disrupted HPV vaccine delivery particularly in 2020 and may have even led to wasted doses. Without appropriately adapting the HPV vaccination programme amid the COVID-19 context, South Africa would have gone on to lose a year's worth of the programme with half a million eligible girls missing out on lifesaving vaccination. Fortunately, the South African National Advisory Group on Immunization recommended pivoting the HPV vaccination programme to prioritize adolescent girls in grade 5, most of whom may have missed their scheduled doses in 2020 due to the pandemic (43). It is anticipated that this critical intervention will reduce missed opportunities for HPV vaccination and thereby minimize the potential long-term consequences on incident HPV infections and associated sequelae as a result of the COVID-19-related disruptions. Maintaining school-based HPV vaccine delivery strategies and catch-up programmes, in combination with intensified social mobilization and community awareness campaigns, as well as ensuring strong provider recommendations and reminders, have all been cited as key interventions for overcoming barriers to HPV vaccination during the COVID-19 era (20, 44).

### Demand-Side Determinants of Programme Performance

Public demand for the HPV vaccine may be influenced by the level of knowledge, awareness and attitudes of the various stakeholders involved in the HPV vaccination programme. These stakeholders include, the parents or caregivers of adolescent girls, adolescent girls themselves, their peers, teachers, and healthcare providers. The influence of these stakeholders on the performance of the vaccination programme stresses the need for sustained, inclusive, and strengthened vaccine communication, education and promotional campaigns aimed at increasing stakeholders' knowledge and awareness about the life-time risk of HPV infection and the risks and benefits of the HPV vaccine in an effort to increase public trust and encourage positive attitudes toward the vaccination programme. Previous systematic reviews (18, 45) have found that public trust in the broader health system and prior experience with other vaccines also tend to influence HPV vaccine acceptance and uptake in African countries.

To improve HPV vaccine acceptance and uptake, the WHO recommends that national immunization programmes invest in effective vaccine communication strategies and provides clear guidance on developing and delivering such strategies (46). In South Africa, healthcare providers consider mothers as the most influential decision-makers in whether adolescent girls receive the HPV vaccine, followed by fathers, adolescent girls themselves, and grandmothers (47). This gives an indication of healthcare providers' primary targets for delivering information and recommendations about the HPV vaccine. The most common interventions used in HPV vaccine communication strategies targeted at adolescents in LMICs include written and oral communication delivered through information fact sheets, radio announcements or programmes, house-to-house education sessions, telephone reminders and messages, and DVD-based instructions (48). Healthcare providers in South Africa find that the most useful sources of information when delivering recommendations about the HPV vaccine to their clients, are television, posters and brochures, flyers, websites, and information handed out at schools, while traditional media sources such as radio and print media, and word-of-mouth are the least useful communication strategies (47).

One of the persistent reasons for reluctance or non-acceptance of the HPV vaccine among stakeholders has been the misconceptions about the safety of the vaccine. Even among parents and caregivers with high levels of education, unaddressed safety concerns about the HPV vaccine may fuel negative attitudes like hesitancy or unacceptance (49). Our review of the published literature from South Africa returned no reports of long-term, vaccine-related serious adverse events either following demonstration projects, clinical trials, or during the nationwide HPV vaccination programme to date, emphasizing the safety of the vaccine (21, 22, 50–52). Commonly reported systemic adverse events following immunization with the HPV vaccine have been consistent with those advised by the WHO (14) and include minor, time-limited incidents of rash, abdominal pain, raised temperature, dizziness, nausea, vomiting, and fainting (21, 22, 51, 52).

Without easy access to careful and transparent information about the safety and benefits of the HPV vaccine, stakeholders may not be able to demand lifesaving HPV vaccines, instead turning to readily available false and often dangerous information in making the decision to refuse the vaccine altogether. Although difficult to quantify, it has been suggested that vaccine hesitancy may play an important role in the suboptimal HPV vaccine coverage and completion rates observed in South Africa, however this is yet to be comprehensively researched (53). Among adolescent girls attending private schools for example, low HPV vaccine uptake has been found to be fueled by misinformation spread via social media platforms which are accessed by their parents and caregivers (29). A recent South African study (54) demonstrated how anti-vaxxers use social media platforms to propagate false information about the risks of HPV vaccination and encourage vaccine hesitancy among parents and caregivers of eligible adolescent girls. This study further identified some determinants of vaccine hesitancy which include misconceptions about the lifetime risk of HPV infection, the effect of the vaccine on reproductive health, the effectiveness of the vaccine, concerns about the informed consenting process, and the use of the school-based strategy for delivering the vaccine (54). Such evidence is crucial and could inform targeted communication strategies that address these concerns and dispel misconceptions by making positive vaccine information readily accessible to all stakeholders and thereby countering anti-vaxxer practices in the South African setting.

Demand generation for the HPV vaccine in South Africa can be improved through continued multi-level engagements with the full scope of stakeholders. Such engagements should adopt tailored, community-based communication and sensitization campaigns with the goal of increasing public awareness and trust in the HPV vaccine. In the current COVID-19 context, clear and effective communication strategies will be important in ensuring that adolescent girls and their parents or caregivers do not confuse ongoing COVID-19 vaccine roll-out with the HPV vaccination programme. The need to empower adolescent girls to demand lifesaving HPV vaccines cannot be overstated. Opportunities exist for creating national spaces and platforms which encourage adolescent girls to be at the forefront of HPV and cervical cancer advocacy and awareness campaigns which can be linked with existing initiatives like the She Conquers campaign (http://sheconquerssa.co.za/) and B-Wise (https://bwisehealth.com/), as well as others with similar mandates.

### High Incidence of HIV Infection Among Adolescent Girls in South Africa

In the South African setting, HIV infected AGYW are a special group of interest given their increased risk for persistent HPV infection, high-grade cervical lesions, and rapid progression to HPV-related invasive cervical disease (8, 55–58). What this implies is that HIV co-infected AGYW may develop cervical cancer at an earlier age, most likely in their prime reproductive and working years, resulting in more years of life lost which has negative implications for productive capacity, as well as economic, societal, and health systems. For AGYW living with HIV, the WHO recommends a three-dose (0, 1–2, 6 months) rather than a two-dose HPV vaccination schedule regardless of whether they are receiving antiretroviral therapy (14).

Although clinical trials (59, 60) have conclusively demonstrated the safety and immunogenicity of HPV vaccines in HIV infected AGYW, a previous modeling study (61) found that the cost-effectiveness of the HPV vaccination programme is lower in the HIV infected sub-population compared to the general female population, requiring a substantial decline in future HIV incidence and mortality rates in South Africa. Unfortunately, when compared to males, the incidence of HIV infection has remained persistently high (up to three-folds higher) among AGYW, a substantial proportion of whom are not on sustained antiretroviral therapy (9, 62). The increased risk of persistent HPV infection and cervical cancer amidst the high incidence of HIV infection in this setting necessitates rapid scale-up of vaccine coverage rates among all adolescent girls <15 years of age. For AGYW between the ages of 15–25 years, the current national policy (12) does not make provision for free cervical cancer screening unless at high risk due to HIV infection or immunosuppression caused by other diseases or treatments, in accordance with global recommendations (10). Unfortunately, uptake of these screening services among HIV infected AGYW in South Africa is reported to be poor, leaving them at high risk of developing invasive cervical cancer (63). This provides further cause for extending the lifesaving benefits of the HPV vaccine to AGYW 15–25 years of age regardless of HIV status, although the feasibility of such an intervention will have to be fully researched. A recent South African study (64) conducted in a setting considered to be the epicenter for both the HIV and cervical cancer epidemics has found that fiscal constraint is the major barrier to accessing the HPV vaccine among AGYW >15 years. Evidently, the unique epidemiology of HPV and HIV infections in South Africa warrants extraordinary interventions if adolescent health is to be prioritized and cervical cancer elimination targets are to be attained.

## Opportunities for Delivering Other Adolescent Health Services Alongside the HPV Vaccine

Although adolescent girls are the only target of the current South African HPV vaccination programme, it is well recognized that both adolescent girls and boys have other healthcare needs and should have access to a full-range of services including deworming, screening for vision and hearing impairment, sexual and reproductive health education, access to contraception and menstrual hygiene products, other adolescent vaccinations, mental healthcare, and counseling and rehabilitation for substance abuse (65). Integrating health services is a cost-effective way of delivering adolescent health services all the while increasing adolescents' interaction with the health system and improving their well-being and quality of life. In this way, delivering other adolescent health services alongside the HPV vaccine can simultaneously improve uptake of these services and vaccine coverage rates (66). Successful delivery of other adolescent health services alongside the HPV vaccine has been reported in other LMICs in sub-Saharan Africa, like Rwanda (67) and Uganda (68).

The Integrated School Health Policy (ISHP) (69) makes provision for a comprehensive adolescent package of healthcare in South Africa. However, it has been reported that implementation of the ISHP has met with several challenges, including non-compliance which negatively impacts on its effectiveness as a platform for optimal delivery of the full complement of school-based adolescent health services (70, 71). Despite this, South Africa has been able to provide services such as deworming and health education in schools alongside the HPV vaccination programme (72).

There is limited evidence in the published literature on the co-delivery of HPV vaccines and other adolescent health services in South Africa. Further operational research studies are required to improve our limited understanding of the successes and challenges of such integrated programmes. Evidence from elsewhere suggests that while delivering the HPV vaccine alongside other adolescent health services in schools offers significant prospects in terms of adolescent health and wellbeing, such programmes come with substantial financial, logistical, and operational challenges (66). In this regard, one of the major concerns with delivering other adolescent health services alongside the HPV vaccination programme lies with the capacity of vaccinators or school health teams – in terms of availability, capability, workload, and staffing – to adequately administer these services during their visits. Watson-Jones et al. (73) previously assessed the feasibility and acceptability of delivering other adolescent health services alongside HPV vaccination in Tanzania and found general support for the intervention among key stakeholders. Similar findings have been reported in the South African context (63). What concerns stakeholders about these integrated approaches aside from the increased workload is the limited availability of infrastructure such as transportation for vaccinators, and the fact that frequent deployment of vaccinators to schools to deliver additional services could have a negative impact on routine health facility visits. Furthermore, delivery of additional services will increase health resource needs and training requirements for vaccinators (73). The potential for disruptions to the busy academic calendar is another important consideration when delivering multiple health services to adolescents in schools. These potential barriers underscore the need for intensively coordinated and adequately resourced integrated services that are designed with careful consideration for local contexts and healthcare priorities.

**Figure 3:**
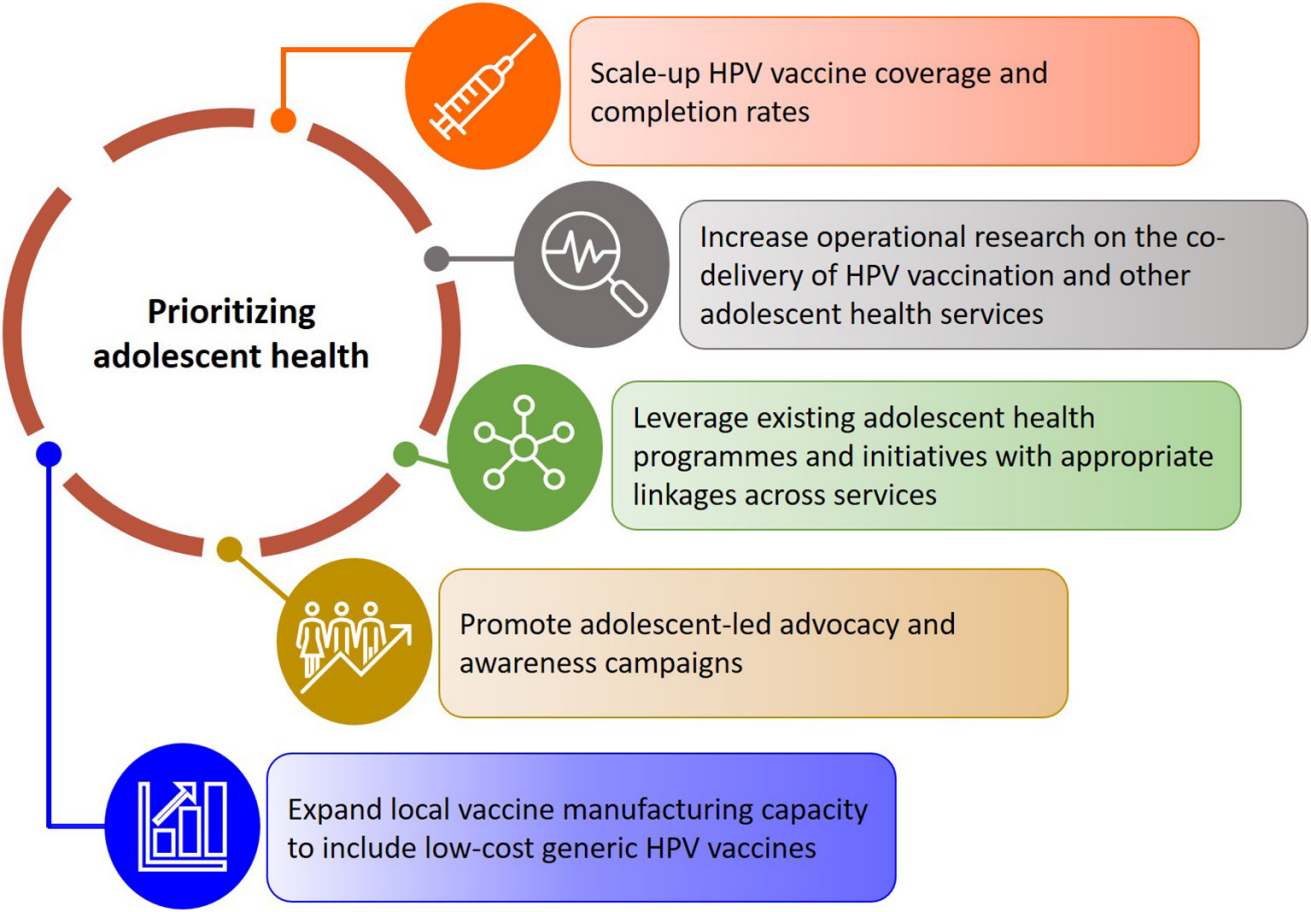
Recommendations for scaling up adolescent health services alongside the HPV vaccination programme in South Africa.

We present recommendations for expanding current research and practice aimed at scaling up the delivery and uptake of adolescent health services alongside the HPV vaccination programme in South Africa (Figure 3). Drawing on the findings of this review, it is evident that integrated adolescent and school-based health programmes in South Africa will have to prioritize strengthening comprehensive sexual and reproductive health and rights education, with a focus on empowering adolescent girls and boys to take ownership of their health and wellbeing. In the short-term, this can be achieved by leveraging existing resources and ensuring appropriate linkages across programmes. While services provided through health and youth facilities, as well as nationally supported campaigns and initiatives like She Conquers and B-Wise exist, and complement relevant courses within the school curriculum, it is important that these programmes are appropriately coordinated to reduce fragmentation of services and maximize the reach of already limited resources. This emphasizes the need for strong collaboration across the various existing programmes including the South African expanded programme on immunization, adolescent health, school health, sexual and reproductive health, women's health, HIV prevention and treatment programmes, and cancer prevention and control initiatives.

## Study Limitations

Although a systematic literature search was performed as part of this narrative review, it is possible that the strategy used may have excluded key literature sources. While we attempted to adequately capture core themes and trends emerging from the pre-HPV vaccine introduction era to date by expanding the scope of the review to include literature sources published between 2010 and 2021, the findings may not comprehensively capture the real-world experiences of the HPV vaccination programme in South Africa. In addition, the review findings may not be generalizable to all settings and calls for further systematic reviews and syntheses to be conducted.

## Conclusion

Overall, sustained implementation of a nationwide school-based HPV vaccination programme in South Africa has been successful, at least over the past 6 years. Moving forward, improving demand generation will be crucial in recovering optimal HPV vaccine coverage and completion rates among eligible adolescent girls. This will have to be supported by up-to-date research evidence on the determinants of HPV vaccine uptake and dose completion. Maximizing the utility of the HPV vaccination programme as an entry point for delivering comprehensive adolescent health care will require, (i) further high-level political commitment and multi-sectoral collaboration, aimed at scaling up the HPV vaccination programme with the local unique disease epidemiology in mind, (ii) reinforcing the implementation of the integrated school health policy, and (iii) appropriately linking adolescents to existing programmes and initiatives which meet their expressed healthcare needs.

As more countries in sub-Saharan Africa prepare to introduce nationwide HPV vaccination programmes, policymakers and immunization managers will rely on the lessons learned in countries with existing and mature programmes. While the South African HPV vaccination programme may be unique for several reasons including the fact that it is fully funded by the national government, the experiences gained over the past 6 years are worth sharing and could make useful contributions to the body of evidence needed to accelerate HPV vaccination and cervical cancer elimination efforts across sub-Saharan Africa. Future HPV vaccination programmes in sub-Saharan Africa will have to secure the involvement of policy makers and influencers, including adolescents, to support and sustain implementation. Furthermore, future, and existing HPV vaccination programmes in sub-Saharan Africa will need to strengthen the implementation of context-specific and adolescent-centered strategies needed to maintain high demand for HPV vaccination and reduce missed opportunities for vaccination and dropout rates. It is also important to stress the need for adaptable HPV vaccination programmes which can withstand acute and prolonged health systems shocks such as those caused by disease outbreaks and pandemics. Finally, given that the widely observed inequitable distribution of COVID-19 vaccines has strengthened calls to invest in scaling up vaccine manufacturing capacity within sub-Saharan Africa, it is imperative that the production of low-cost generic HPV vaccines form part of this regional policy agenda as an avenue to address vaccine supply challenges.

## Author Contributions

EA-D developed the review concept and wrote the initial draft of the manuscript. EA-D, NB, VVN, and VC contributed to the overall review process, including the literature search, screening, data extraction, and analysis. All authors approved the final version of the manuscript.

## Funding

EA-D was supported by funding from the Harry Crossley Postdoctoral Research Fellowship, 2021. The funder had no role in the conception of the review, data collection and analysis, preparation of the manuscript, or the decision to publish.

## Conflict of Interest

The authors declare that the research was conducted in the absence of any commercial or financial relationships that could be construed as a potential conflict of interest.

## Publisher's Note

All claims expressed in this article are solely those of the authors and do not necessarily represent those of their affiliated organizations, or those of the publisher, the editors and the reviewers. Any product that may be evaluated in this article, or claim that may be made by its manufacturer, is not guaranteed or endorsed by the publisher.
